# Oral Epithelial Cells Expressing Low or Undetectable Levels of Human Angiotensin-Converting Enzyme 2 Are Susceptible to SARS-CoV-2 Virus Infection In Vitro

**DOI:** 10.3390/pathogens12060843

**Published:** 2023-06-19

**Authors:** Laith Ebraham, Chuan Xu, Annie Wang, Cyril Hernandez, Nicholas Siclari, Divino Rajah, Lewins Walter, Salvatore A. E. Marras, Sanjay Tyagi, Daniel H. Fine, Carlo Amorin Daep, Theresa L. Chang

**Affiliations:** 1 Public Health Research Institute, New Jersey Medical School, Rutgers, The State University of New Jersey, Newark, NJ 07103, USA; 2 Global Technology Center, Colgate-Palmolive Company, Piscataway, NJ 08855, USA; 3 Department of Microbiology, Biochemistry and Molecular Genetics, New Jersey Medical School, Rutgers, The State University of New Jersey, Newark, NJ 07103, USA; 4 Department of Oral Biology, School of Dental Medicine, Rutgers, The State University of New Jersey, Newark, NJ 07103, USA

**Keywords:** SARS-CoV-2, oral epithelial cells

## Abstract

The oral cavity is thought to be one of the portals for SARS-CoV-2 entry, although there is limited evidence of active oral infection by SARS-CoV-2 viruses. We assessed the capacity of SARS-CoV-2 to infect and replicate in oral epithelial cells. Oral gingival epithelial cells (hTERT TIGKs), salivary gland epithelial cells (A-253), and oral buccal epithelial cells (TR146), which occupy different regions of the oral cavity, were challenged with replication-competent SARS-CoV-2 viruses and with pseudo-typed viruses expressing SARS-CoV-2 spike proteins. All oral epithelial cells expressing undetectable or low levels of human angiotensin-converting enzyme 2 (hACE2) but high levels of the alternative receptor CD147 were susceptible to SARS-CoV-2 infection. Distinct viral dynamics were seen in hTERT TIGKs compared to A-253 and TR146 cells. For example, levels of viral transcripts were sustained in hTERT TIGKs but were significantly decreased in A-253 and TR146 cells on day 3 after infection. Analysis of oral epithelial cells infected by replication-competent SARS-CoV-2 viruses expressing GFP showed that the GFP signal and SARS-CoV-2 mRNAs were not evenly distributed. Furthermore, we found cumulative SARS-CoV-2 RNAs from released viruses in the media from oral epithelial cells on day 1 and day 2 after infection, indicating productive viral infection. Taken together, our results demonstrated that oral epithelial cells were susceptible to SARS-CoV-2 viruses despite low or undetectable levels of hACE2, suggesting that alternative receptors contribute to SARS-CoV-2 infection and may be considered for the development of future vaccines and therapeutics.

## 1. Introduction

The first case of coronavirus disease (COVID-19) was reported in December 2019, and the World Health Organization (WHO) declared the 2019 Novel Coronavirus outbreak a Public Health Emergency of International Concern on 31 January 2020 [1]. Recently, the WHO and Centers for Disease Control and Prevention (CDC) marked the end of the COVID-19 public health emergency in May 2023 [2,3]. However, COVID still remains a threat due to continuously evolving new variants [4,5]. Fully vaccinated people are not devoid of risk for infection by variants of the virus [6,7,8,9].

Severe acute respiratory syndrome coronavirus-2 (SARS-CoV-2), the causative agent for COVID-19, is an enveloped, positive single-stranded RNA virus, and its main entry is mediated by the binding of the SARS-CoV-2 spike protein (RBD) to the human angiotensin-converting enzyme 2 (hACE2) receptor on the cell surface [10,11]. The RBD is the major target for anti-spike neutralizing antibodies in response to infection and vaccination and has been used for therapeutics [10,12]. The viral entry involves viral attachment, endocytosis, and membrane fusion [10]. SARS-CoV-2 spike proteins are cleaved into S1 and S2 subunits by proprotein convertases, such as furin in virus-producing cells [10]. The S1 subunit includes the N-terminal domain and RBD, whereas the S2 subunit promotes membrane fusion. In addition to hACE2 receptors, several hACE2-dependent accessory receptors have been identified that promote SARS-CoV-2 entry. These accessory receptors/molecules include furin [13,14], transmembrane serine protease 2 (TMPRSS2) and TMPRSS4 [11,15], trypsin [16], neuropilin-1 [17,18], cathepsins [19,20], sialic acid-containing glycolipids [21], vimentin [22,23], heparan sulfate [24], and phosphatidylserine receptor [25]. hACE2-mediated viral entry contributes to productive viral infection; however, hACE2-independent SARS-CoV-2 entry has been documented, despite some controversy, and may play a role in altering immune and cell functions (reviewed in [26]). Indeed, analysis of the Alpha, Beta, and Delta SARS-CoV-2 variant spike proteins indicates an altered cell interaction with a reduced dependence on hACE2 [27]. We have previously demonstrated that SARS-CoV-2 infection of Calu-3 lung epithelial cells and CaCo-2 intestinal epithelial cells, both of which express high-abundance hACE2, was sensitive to anti-spike RBD Ab, whereas infection of A459 cells expressing very-low-abundance hACE2 but high levels of CD147 was resistant to anti-spike RBD Ab [28]. The naturally occurring mutation, E484D, in spike proteins allows SARS-CoV-2 to enter cells via an hACE2-independent mechanism, and this entry is resistant to Imdevimab, an antibody used for COVID-19 therapy [29]. Interestingly, heavily mutated Omicron variants exhibit strong binding to hACE2 but escape several approved COVID-19 therapeutic antibodies [30,31]. The highly transmissible Omicron BA.1 variant has largely lost dependence on the protease TMPRSS2 resulting in the use of different entry pathways, a shift in cell tropism, and altered pathogenesis [32,33,34,35]. It is possible that alternative receptors or accessory receptors may contribute to viral evolution and immune escape. Considering that SARS-CoV-2 will likely continue to infect humans regularly, identifying target cells will provide insights relevant to the development of strategies for prevention and therapy.

Transmission of SARS-CoV-2 is known to occur through infected secretions, such as respiratory droplets and aerosol transmission [36]. Due to the mouth’s proximity to the upper respiratory tract, salivary SARS-CoV-2 RNAs and proteins are detectable in infected symptomatic and asymptomatic individuals [37,38,39]. However, direct evidence supporting the oral cavity as a portal of SARS-CoV-2 entry is limited.

Single-cell RNAseq analysis of oral tissues showed that hACE2 can be detected in various oral epithelial cell clusters; although, abundance is low except in salivary ducts [40]. Low numbers of hACE2-expressing cells are detected in the buccal, tongue, and tonsil mucosa, suggesting that multiple sites in the oral cavity and oropharynx are potentially susceptible to infection by SARS-CoV-2 [40]. In this regard, Xu et al. detected hACE2 expression in oral tissues, observing higher abundance in the tongue than in buccal and gingival tissues [41]. Coincidentally, SARS-CoV-2 transcripts were detectable in salivary glands and in various mucosal sites (dorsal tongue, tonsil, and uvula) of COVID-19 autopsy tissues [40]. In addition to being detected in saliva, SARS-CoV-2 RNA was also detected in gingival crevicular fluid and oral plaque [42,43]. In addition, SARS-CoV-2 spike proteins were observed histologically in cytological smears collected from the dorsum of the tongue of COVID-19 patients [44]. We have previously shown that TR146 cells, an oral epithelial cell line mimicking human buccal epithelium, are susceptible to pseudotyped viruses expressing SARS-CoV-2 spike proteins [45]; however, the susceptibility of TR146 cells and other oral epithelial cells, including salivary gland and gingival epithelial cells, to replication-competent viruses is not known. In this study, we determined the susceptibility of oral gingival epithelial cells, submaxillary salivary gland epithelial cells, and buccal oral epithelial cells to SARS-CoV-2.

## 2. Materials and Methods

### 2.1. Cell Culture

HEK293T, Caco-2, Vero, oral gingival epithelium hTERT TIGKs (Telomerase Immortalized Gingival Keratinocytes, ATCC^®^ CRL-3397), and submaxillary salivary gland A-253 (ATCC^®^ HTB-41) cells were purchased from the American Type Cell Collection (ATCC, Manassas, VA, USA). TR146 cells were purchased from Millipore Sigma. HEK293T, Caco-2, Vero, and hACE2-expressing HeLa cells (kindly provided by Dennis Burton; The Scripps Research Institute, La Jolla, CA, USA) were cultured in Dulbecco’s Modified Eagle’s Medium (DMEM) supplemented with 10% fetal bovine serum (FBS). TR146 cells were cultured in Ham’s F12 media with glutamate and 10% FBS. hTERT TIGKs were cultured in dermal cell basal medium (ATCC, PCS-200-030) with keratinocyte growth kits (ATCC, PCS-200-040). A-253 cells were cultured in McCoy’s 5a medium with 10% FBS.

### 2.2. Viral Infection Assay

A replication-defective HIV-1 luciferase-expressing reporter virus pseudotyped with SARS-CoV-2 S proteins was produced by co-transfection of a plasmid encoding the envelope-deficient HIV-1 NL4-3 virus with the luciferase reporter gene (pNL4-3.Luc.R+ E-, kindly provided by Nathaniel Landau, New York University, NY, USA) and a pcDNA3.1 plasmid expressing the SARS-CoV-2 glycoprotein from the Wuhan strain [28] or a plasmid expressing Omicron XBB1.5 (kindly provided by Dennis Burton, The Scripps Research Institute, La Jolla, CA, USA) into HEK 293T cells using Lipofectamine 3000 (Thermo Fisher Scientific, Waltham, MA, USA) as described previously [28]. The supernatant was collected 48 h after transfection and filtered. Virus stocks were analyzed for HIV-1 p24 antigen by the AlphaLISA HIV p24 kit (PerkinElmer, Waltham, MA, USA). Virus stocks contained approximately 200 ng/mL of HIV p24 proteins. Cells were seeded at 5 × 10^4^ cells/well in a 48-well plate, cultured overnight, and infected with pseudotyped SARS-CoV-2 luciferase reporter virus. After 1–2 h viral attachment, infected cells were cultured in media with 10% FBS for 48–72 h. Cells were then lysed in 1× passive lysis buffer (Promega Inc., Madison, WI, USA), and luciferase activity (relative light units; RLUs) was measured using Luciferase Substrate Buffer (Promega Inc.) on a 2300 EnSpire Multilabel Plate Reader (PerkinElmer).

The replication-competent SARS-CoV-2 USA-WA1/2020 strain (BEI Resources, ATCC, Manassas, VA, USA) or SARS-CoV-2 Wuhan strain expressing mNeonGreen were propagated in Vero E6 cells as described previously [46]. Virus propagation was performed in a Biosafety Level 3 laboratory with personal protection equipment, including powered air-purifying respirators (Breathe Easy, 3M, Saint Paul, MN, USA), Tyvek suits, aprons, sleeves, booties, and double gloves. Titers of our replication-competent virus stocks were determined using plaque assays in Vero E6 cells as described previously [28].

### 2.3. Flow Cytometry

To determine cell surface expression of hACE2, CD147, and AXL, oral epithelial cells and appropriate positive control cell lines were collected in PBS using cell scrapers. Cells were blocked in wash buffer (PBS, 2% FBS, 1% human serum AB) on ice for 20 min and then stained with fluorochrome-conjugated specific antibodies against hACE2 (R&D Systems, Minneapolis, MN, USA, cat #FAB933A100), CD147 (AnCell Crop, Bayport, MN, USA, cat# 376-040), or AXL (Invitrogen, Waltham, MA, USA, cat #17108742) and with appropriate isotype controls in wash buffer for 20 min. Cells were washed, fixed with 2% paraformaldehyde in PBS, and analyzed using a BD Bioscience LSRII flow cytometer (BD Biosciences, San Jose, CA, USA). Results were analyzed with FlowJo (Tree Star, Inc., Ashland, OR, USA).

### 2.4. Real-Time RT-Quantitative PCR Analysis

To determine viral RNAs in cells, total RNA was isolated using TRIzol^®^ (Life Technologies, Carlsbad, CA, USA) followed by Zymo RNA isolation kits. To determine the level of viruses in the culture media from infected cells, viral RNAs were isolated using QIAmp Viral RNA kits (Qiagen, Valencia, CA, USA). The SARS-CoV-2 RNA-dependent RNA polymerase gene (RdRP) and the SARS-CoV-2 nucleocapsid gene (N), together with the human β-actin gene as an internal control were quantified using RT-qPCR as described recently [47]. The reactions were carried out in a 20 µL volume that contained 1× TaqPath 1-step RT-qPCR Master Mix (A28521, Thermo Fisher Scientific), 100 nM CoV-RdRP forward primer, 500 nM CoV-RdRP reverse primer, 250 nM CoV-RdRP molecular beacon probe, 100 nM CoV-N forward primer, 500 nM CoV-N reverse primer, 250 nM CoV-N molecular beacon probe, 100 nM β-actin forward primer, 500 nM β-actin reverse primer, and 250 nM β-actin molecular beacon probe. Total RNAs (1.5 µg) were used in each reaction. The RT-PCR assays were performed in 200 µL white polypropylene PCR tubes (USA Scientific, Ocala, FL, USA) in a CFX96 Touch real-time PCR Detection System (Bio-Rad Laboratories, Hercules, CA, USA). The thermal cycler was programed to incubate the reaction mixtures for 10 min at 53 °C to generate cDNA, followed by 2 min at 95 °C to activate the DNA polymerase and by 45 thermal cycles that consisted of DNA denaturation at 95 °C for 15 s and primer annealing and elongation at 58 °C for 30 s. Molecular beacon fluorescence intensity was monitored during the 58 °C annealing and chain elongation stage of each thermal cycle. The primers and molecular beacons (Table 1) were purchased from LGC Biosearch Technologies (Middlesex, UK), and their use was modified and adapted from a previously validated assay [48].

To determine the receptors for SARS-CoV-2, first-strand cDNA was synthesized by incubating 1000 ng total RNA with oligo(dT) (25 μg/mL) and dNTP (0.5 mM) at 65 °C for 5 min followed by quick-chilling on ice. RT was performed at 42 °C for 50 min and 70 °C for 15 min using SuperScript III Reverse Transcriptase. The PCR assay contained cDNA equivalent to 30 ng of RNA input, 200 nM primer sets, and SYBR Green Master Mix (QIAGEN) and was run in a StepOnePlus real-time PCR system (Life Technologies, Carlsbad, CA, USA). PCR conditions included 95 °C denaturation for 10 min, 40 cycles of 95 °C for 15 s and 60 °C for 60 s. PCR products were quantified and normalized relative to the amount of GAPDH cDNA product. Relative quantification of gene expression was calculated using the ΔΔCt (Ct, threshold cycle of real-time PCR) method according to the following formulas: ΔCT = Ct GAPDH – Ct target, ΔΔCt = ΔCt control – ΔCt target. Primer sequences are listed in Table 2.

### 2.5. Single-Molecule Fluorescence In Situ Hybridization (smFISH)

smFISH for SARS-CoV-2 RNA was performed following procedures described previously [49]. We designed a total of 336 3′-amino labeled oligonucleotide probes for the SARS-CoV-2 genomic RNA using Stellaris Probe Designer employing the highest stringency settings for the human host, pooled all probes, coupled them to Cy5, and then purified them using HPLC [50]. Cells were cultured overnight on coverslips coated with 0.1% gelatin in 12-well plates and then exposed to SARS-CoV-2 viruses expressing GFP at a multiplicity of infection (MOI) of 0.5 for 1 h. At 24 and 48 h post-infection, cells on the coverslips were washed with PBS and then fixed with 10% formaldehyde in 1X PBS for 10 min, washed with PBS, and stored in 70% ethanol at −20 °C. The coverslips were equilibrated with 2XSSC, 10% formamide (wash buffer) and then hybridized in 50 µL hybridization buffer supplemented with 25 ng of pooled probes overnight at 37 °C in a humid chamber. The coverslips were washed twice for 5 min with wash buffer, equilibrated with 2XSSC supplemented with 0.4% glucose, and then mounted using deoxygenated mounting medium supplemented with DAPI [49]. Approximately 20 images per sample were acquired using a Zeiss Axiovert M200 microscope with a 20× objective.

### 2.6. Statistical Analysis

Statistical comparisons were performed using one-way ANOVA Dunnett’s multiple comparisons test or two-tailed Mann–Whitney U test as appropriate. Prism 8 (GraphPad Software, LLC, San Diego, CA, USA) was used. *p* < 0.05 was considered significant.

## 3. Results

### 3.1. Expression of hACE2 and Alternative Receptors CD147 and AXL

**Figure 1:**
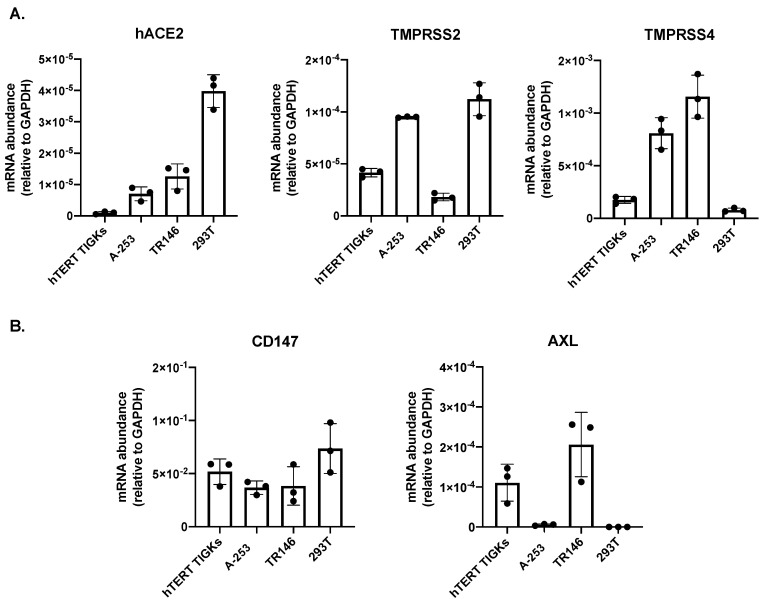
Expression of hACE2, TMPRSS2, TMPRSS4, CD147, and AXL mRNA in oral epithelial cell lines. Total RNAs were extracted from oral gingival epithelial cells (hTERT TIGKs), salivary gland epithelial cells (A-253), oral buccal epithelial cells (TR146) and HEK293T cells. (**A**) Expression of hACE2, TMPRSS2, and TMPRSS4. (**B**) Expression of hACE2-independent alternative receptors CD147 (left) and AXL (right). GAPDH was used for normalization. Experiments were conducted in triplicate. Each dot represents one data point. The result represents two independent experiments.

hACE2 has been considered to be a determinant of SARS-CoV-2 cell and tissue tropism via the receptor-binding domain (RBD) of the surface/spike protein. Additionally, transmembrane protease serine 2 (TMPRSS2) and 4 (TMPRSS4) interact with SARS-CoV-2 spike proteins and promote hACE2-mediated viral entry [11,15]. We determined the expression of hACE2, TMPRSS2, and TMPRSS4 mRNA in oral gingival epithelial cells (hTERT TIGKs), submaxillary salivary gland A-253 cells, and oral buccal epithelial TR146 cells. HEK293T cells, which were used in our previous analyses for hACE2 expression in various cell lines and which were not permissive for SARS-CoV-2 [28], were included for comparison. All oral epithelial cells analyzed in this study expressed low levels of hACE2 mRNAs (Figure 1A). In hTERT TIGKs, hACE2 expression was nearly undetectable. Gene expression profiles of TMPRSS2 and TMPRSS4 varied among oral epithelial cells. A-253 cells expressed both TMPRSS2 and TMPRSS4, whereas hTERT TIGKs had detectable TMPRSS2 but low levels of TMPRSS4. TR146 cells had a low abundance of TMPRSS2 but a high abundance of TMPRSS4.

Alternative receptors for SARS-CoV-2 viral entry, including CD147 (known as *basigin* or extracellular matrix metalloproteinase inducer, EMMPRIN) and AXL (tyrosine-protein kinase receptor UFO), have also been reported, particularly in cells with low-abundance hACE2 [28,51,52]. We have previously shown that SARS-CoV-2 infects cells with a low abundance of hACE2 via CD147 in a spike RBD-independent manner [28]. RT-qPCR analyses of CD147 and AXL mRNAs showed that all oral epithelial cells expressed high levels of CD147 (Figure 1B). AXL mRNAs were expressed in hTERT TIGKs and TR146 cells but were nearly undetectable in A-253 cells.

**Figure 2:**
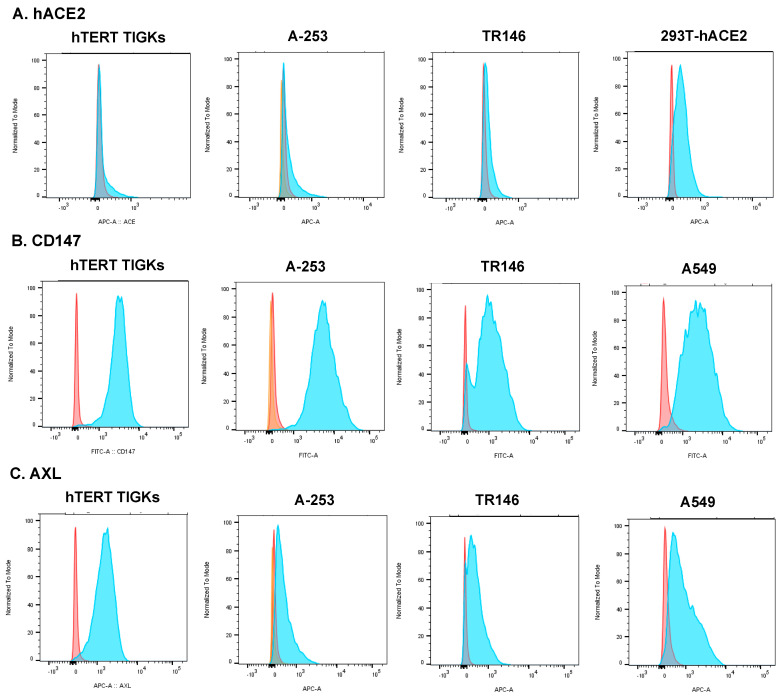
Cell surface protein expression of hACE2, CD147, and AXL mRNA in oral epithelial cell lines. Oral gingival epithelial cells (hTERT TIGKs), salivary gland epithelial cells (A-253), and oral buccal epithelial cells (TR146) were analyzed using flow cytometry. 293T-hACE2 cells were included as a positive control for hACE2, and A549 cells were used as controls for CD147 and AXL. The signals from isotype control antibodies (orange) and antibodies targeting specific receptors (blue) are shown. The result is representative of two independent experiments.

Cell surface protein expression of hACE2, CD147, and AXL on hTERT TIGKs and TR146 cells was also analyzed using flow cytometry. 293T-hACE2 and A549 cells were included as controls for hACE2 and alternative receptors, respectively (Figure 2). In agreement with the RT-qPCR result, the level of protein expression of hACE2 was low in all tested oral epithelial cells, whereas the level of CD147 proteins was high. The level of cell surface AXL expression was high in hTERT TIGKs followed by TR146 cells and A-253 cells.

### 3.2. Various Oral Epithelial Cell Lines Are Susceptible to SARS-CoV-2

**Figure 3:**
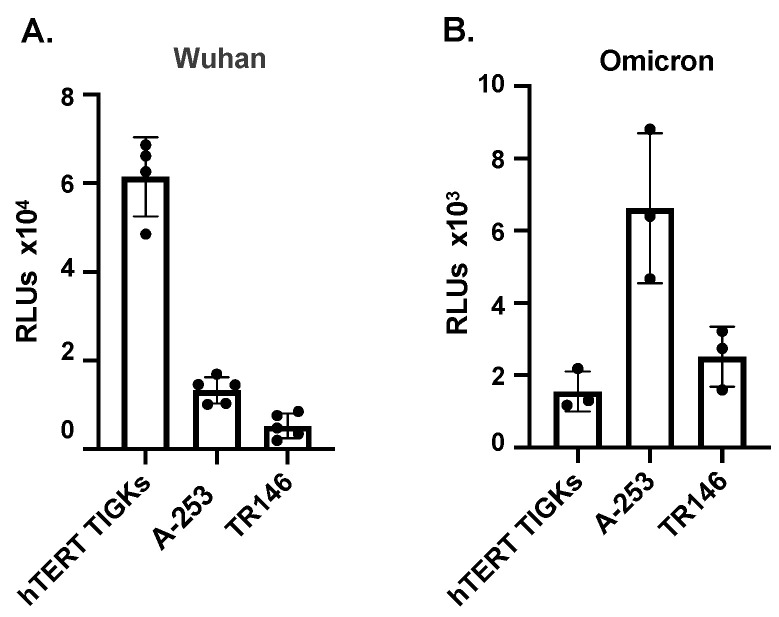
Infection of oral epithelial cells by pseudotyped SARS-CoV-2 viruses expressing spike proteins from the Wuhan strain and the XBB1.5 omicron subvariant. Oral gingival epithelial cells (hTERT TIGKs), salivary gland epithelial cells (A-253), and oral buccal epithelial cells (TR146) were infected by pseudotyped viruses expressing SARS-CoV-2 spike proteins from the Wuhan strain (**A**) and omicron variant (**B**). Infection was determined by measuring luciferase activity at 72 h after infection. Each sample contained 3–4 replicates. Data are shown as mean ± SD. The result is representative of three independent experiments.

To determine whether oral epithelial cells representing different sites of the oral cavity were susceptible to SARS-CoV-2, pseudotyped luciferase reporter viruses expressing SARS-CoV-2 spike proteins from the Wuhan strain (Figure 3A) and the omicron XBB1.5 variant (Figure 3B) were tested in the viral entry assay. hTER TIGKs, A-253, and TR146 cells were susceptible to the SARS-CoV-2 pseudotyped virus (Figure 3), but the degree of infection did not correlate with hACE2 expression (Figure 1A and Figure 2), suggesting the possible involvement of alternative receptors or other accessory factors in viral entry.

**Figure 4:**
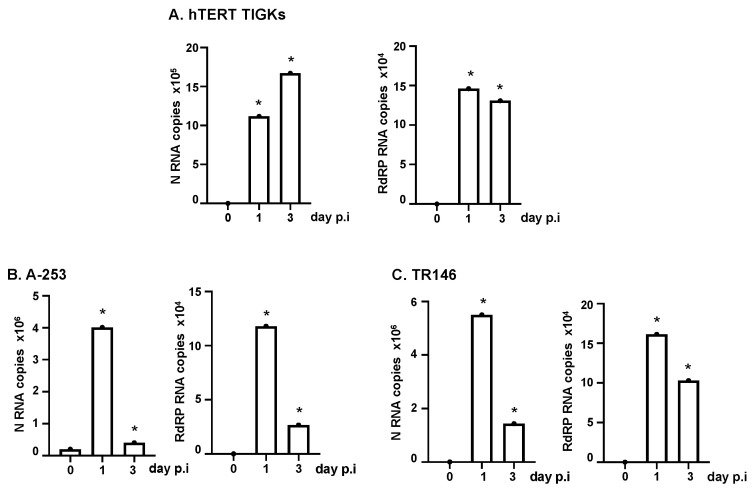
Infection of oral epithelial cells by replication-competent SARS-CoV-2 WA 01/2020 strain. Oral gingival epithelial cells (hTERT TIGKs) (**A**), salivary gland epithelial cells (A-253) (**B**), and oral buccal epithelial cells (TR146) (**C**) were exposed to replication-competent SARS-CoV-2 WA 01/2020 strain at an MOI of 0.5 for 1 h, washed with PBS, and placed in culture. Total RNAs were prepared at 2 h (day 0), day 1, and day 3 p.i. SARS-CoV-2 N and RdRP RNA copy numbers and β-actin were analyzed using RT-qPCR. Copy numbers from approximately 1 × 10^5^ cells are shown. Data are mean ± SD; * *p* < 0.05, infected samples vs. the day 0 sample. The result is representative of two independent experiments.

We then infected oral epithelial cells with replication-competent SARS-CoV-2 WA strains at MOI 0.5. Total RNAs from infected cells were prepared at 2 h, 1 day, and 3 days post-infection (p.i.). SARS-CoV-2 N (nucleocapsid) and RdPR (RNA-dependent RNA polymerase) transcripts were determined using RT-qPCR analysis. There was an increase in both SARS-CoV-2 transcripts on day 1 compared to day 0 (2 h) p.i., indicating productive viral replication. At day 3 p.i., the levels of viral transcripts were sustained in hTERT TIGKs but were significantly decreased in A-253 and TR146 cells (Figure 4A–C). Note that viral transcripts among the total RNA of the population of infected cells were measured; thus, reduction in viral mRNA was unlikely due to virus-induced cell death. Indeed, we did not observe apparent cell death (e.g., rounded-up cells) of oral epithelial cells in response to SARS-CoV-2 infection (Supplementary Figure S2). Interestingly, SARS-CoV-2 altered the cell morphology of oral epithelial cells despite the fact that changes were subtle (Supplementary Figure S2).

**Figure 5:**
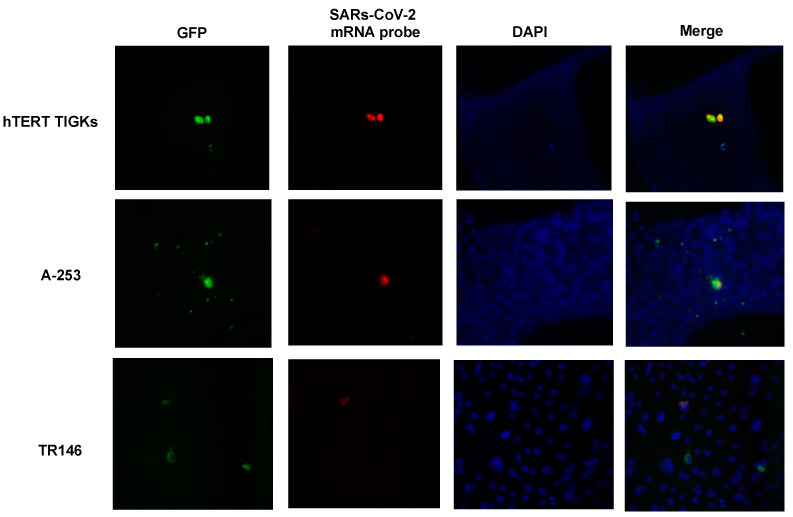
Infection of oral epithelial cells by replication-competent SARS-CoV-2 Wuhan strain expressing GFP. Oral gingival epithelial cells (hTERT TIGKs), salivary gland epithelial cells (A-253), and oral buccal epithelial cells (TR146) were infected by SARS-CoV-2 Wuhan strain expressing GFP. Infection was determined using detecting fluorescence (GFP) and smFISH with SARS-CoV-2 mRNA probes at 48 h p.i. Nuclei were stained with DAPI. Images were acquired on a Zeiss Axiovert M200 microscope with a 20× objective. The result is representative of two independent experiments.

We then validated the susceptibility of oral epithelial cells to SARS-CoV-2 using microscopy. Oral epithelial cells were infected with replication-competent SARS-CoV-2 viruses expressing GFP. In addition to visualizing GFP signals, SARS-CoV-2 RNAs were also analyzed using smFISH. Overall, the signals of GFP and SARS-CoV-2 mRNAs were co-localized in the same cells (Figure 5). Both GFP proteins and SARS-CoV-2 RNAs were detectable at 24 h p.i., and the signals were not increased at 48 h p.i. (data not shown for 24 h p.i.). Neither GFP nor SARS-CoV-2 signals were detectable in uninfected cells (Supplementary Figure S1). Regardless of the type of oral epithelial cells, the viral signal was low in most cells. However, a few cells, typically one cell per field of view, had high viral signals. We observed mostly single cells with viral mRNAs rather than two adjacent infected cells, suggesting the absence of cell-cell viral transmission. Occasionally, we observed two infected cells that were adjacent to each other, which could arise due to cell division after infection. We conclude that hTER TIGKs, A-253, and TR146 cells were susceptible to SARS-CoV-2 (Figure 3, Figure 4 and Figure 5) but the viral dynamics in hTER TIGKs differed from those in A-253 and TR146 cells, as RNA levels were maintained in hTER TIGKs (Figure 4A) but decreased rapidly from day 1 to day 3 in A-253 (Figure 4B) and TR146 cells (Figure 4C).

**Figure 6:**
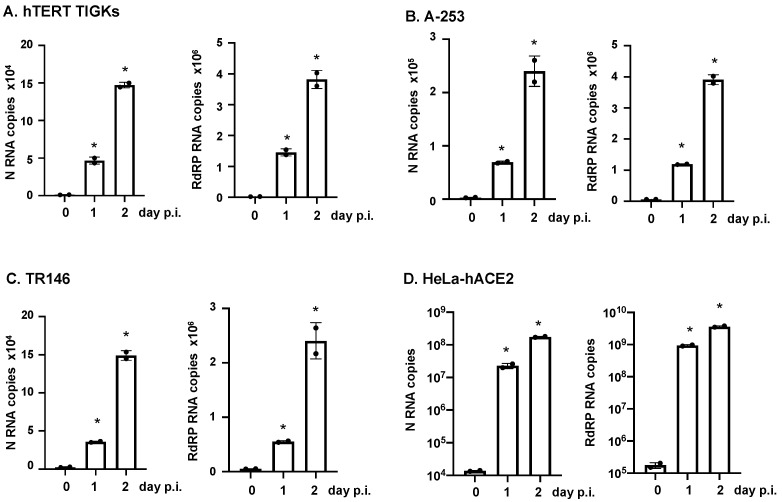
Infection of oral epithelial cells by replication-competent SARS-CoV-2 WA 01/2020 strain. Oral gingival epithelial cells (hTERT TIGKs) (**A**), salivary gland epithelial cells (A-253) (**B**), and oral buccal epithelial cells (TR146) (**C**) were exposed to replication-competent SARS-CoV-2 WA 01/2020 strain at an MOI of 0.5 for 1 h, washed with PBS, and placed in culture. HeLa-hACE2 cells **(D**) were also infected with SARS-CoV-2 WA 01/2020 strain as a comparison. Total RNAs were prepared on day 0 (2 h), day 1, and day 3 p.i. SARS-CoV-2 N and RdRP RNA copy numbers and β-actin were analyzed using RT-qPCR. Copy numbers from approximately 1 × 10^5^ cells are shown. Data are mean ± SD; * *p* < 0.05, infected samples vs. the day 0 sample. The result is representative of two independent experiments.

To determine whether productive SARS-CoV-2 infection occurred in oral epithelial cells after infection, we measured the levels of viral RNAs from released viruses in the culture media at different time points after infection. Culture media from infected cells were collected 2 h after viral attachment (baseline) and on days 1 and 2 p.i., and SARS-CoV-2 N and RdPR RNAs were analyzed using RT-PCR. HeLa-hACE2 cells were included as a positive control (Figure 6). There was a significant increase in viral RNA levels between the baseline (day 0) and day 1 in the culture media, indicating productive viral infection. We also found cumulative SARS-CoV-2 RNAs in the media from oral epithelial cells on day 1 and day 2 after infection, indicating productive viral infection. As expected, viral RNA levels in oral epithelial cells were lower than the levels in HeLa-hACE2 cells, supporting a primary role of hACE2 for viral entry.

## 4. Discussion

The oral cavity is considered a gateway for many pathogens, including SARS-CoV-2, which can infect the human body; however, there is limited evidence supporting SARS-CoV-2 replication in oral epithelial cells. Here, we found that oral gingival epithelial cells (hTERT TIGKs), salivary gland epithelial cells (A-253), and oral buccal epithelial cells (TR146) were susceptible to infection by both a pseudotyped virus expressing SARS-CoV-2 spike proteins and by replication-competent SARS-CoV-2.

Assays for SARS-CoV-2 infection of oral epithelial cells have mainly relied on the detection of viral transcripts or proteins in oral tissues from COVID-19 patients. Here, in experiments using a variety of SARS-CoV-2 virus forms, including pseudotyped viruses expressing SARS-CoV-2 spike proteins (for viral entry), replication-competent SARS-CoV-2 viruses, and SARS-CoV-2 viruses expressing GFP, we showed that SARS-CoV-2 infected oral gingival epithelial cells (hTERT TIGKs), salivary gland epithelial cells (A-253), and oral buccal epithelial cells (TR146). Viral transcripts in infected cells were detected using RT-qPCR and smFISH. Additionally, we also found cumulative RNA levels in culture medial from oral epithelial cells. In agreement with previous results in oral tissues from COVID patients [40], a few cells expressed high levels of SARS-CoV-2 mRNAs and GFP proteins. The viral signal remained high at day 3 p.i. in hTERT TIGKs but decreased significantly by day 3 p.i. in A-253 and TR146 cells. SARS-CoV-2 N mRNA copy numbers in A-253 and TR146 were higher than in hTERT TIGKs (4–5.5 × 10^6^ vs. 1 × 10^6^ copies/10^5^ cells). hTERT TIGKs, which are telomerase immortalized cells, were also less transformed than A-253 and TR146, which are tumor-derived cell lines. It remains to be determined whether the cells derived from different sites within the oral cavity or the degree of tumorgenicity contribute to differential infection profiles. It is worth noting that all oral epithelial cells expressed low levels of hACE2 mRNAs and proteins, but the hACE2 level did not correlate with the extent of pseudotyped SARS-CoV-2 virus entry, suggesting the possible involvement of alternative receptors or accessory factors in viral entry. All oral epithelial cells expressed highly abundant CD147, an alternative receptor, and hTERT TIGKs; A-235 cells also expressed AXL, another alternative receptor. SARS-CoV-2 viral entry via CD147 and AXL is known to be independent of hACE2 spike RBD proteins [28,52], which are the targets of current vaccines. In addition to CD147 and AXL, other hACE2-independent receptors for SARS-CoV-2 have been reported (reviewed in [26]). Further studies on expression profiles of alternative receptors and specific receptors and factors promoting SARS-CoV-2 viral entry into oral epithelial cells, as well as studies on virus-mediated alterations of oral epithelial cell functions, will provide a better understanding of SARS-CoV-2 pathogenesis in the oral cavity.

## Figures and Tables

**Table 1:** Primers and molecular beacon probes used in SARS-CoV-2 RT-PCR assay.

Oligonucleotide	Sequence 5′ → 3′
CoV-RdRP forward	GTGARATGGTCATGTGTGGCGG
CoV-RdRP reverse	CARATGTTAAASACACTATTAGCATA
CoV-RdRP molecular beacon	FAM - CGCAG GGTGGAACCTCATCAGGAGATGC CTGCG - BHQ-1
CoV-N forward	GACCCCAAAATCAGCGAAAT
CoV-N reverse	TCTGGTTACTGCCAGTTGAATCTG
CoV-N molecular beacon	CFR - CGCGAG ACCCCGCATTACGTTTGGTGGACC CTCGCG - BHQ-2
β-actin forward	CCCAGCACAATGAAGATCAAGATC
β-actin reverse	AAGCATTTGCGGTGGACGAT
β-actin molecular beacon	Q705 - CGCCCG GCAAGCAGGAGTATGACGAGTCCGG CGGGCG - BHQ-2

FAM = fluorescein, CFR = Cal Fluor Red 610, Q705 = Quasar 705, BHQ = Black Hole Quencher, underlined nucleotides in molecular beacon probes indicate the 5′ and 3′ arm regions of the probe.

**Table 2:** Primers used for RT-qPCR assays for host genes.

Primer	Forward	Reverse
Human GAPDH	5′- GCACCACCAACTGCTTAGCAC-3′	5′-TCTTCTGGGTGGCAGTGATG-3′
Human ACE2	5′-CGAAGCCGAAGACCTGTTCTA-3′	5′-GGGCAAGTGTGGACTGTTCC-3′
Human TMPRSS2	5′-CAAGTGCTCCAACTCTGGGAT-3′	5′- AACACACCGATTCTCGTCCTC-3′
Human TMPRSS4	5′- CCAAGGACCGATCCACAC T-3′	5′- GTGAAGTTGTCGAAACAGGCA-3′;
Human CD147	5′-GTC TTC CTC CCC GAG CCC-3′	5′-GGTGGCACGGACTCTGAC-3′
Human AXL	5′-GTGGGCAACCCAGGGAATATC-3′	5′-GTACTG TCCCGTGTCG GAAAG-3′

## Data Availability

Not applicable.

## Supplementary Materials

The following supporting information can be downloaded at: https://www.mdpi.com/article/10.3390/pathogens12060843/s1. Figuer S1: smFISH analysis of uninfected oral epithelial cells. Figuer S2: Brightfield images of oral epithelial cells with or without SARS-CoV-2 infection.
