# Supplementary material for: Oral Epithelial Cells Expressing Low or Undetectable Levels of Human Angiotensin-Converting Enzyme 2 Are Susceptible to SARS-CoV-2 Virus Infection In Vitro
Source: Pathogens. 2023 Jun 19;12(6):843. doi: 10.3390/pathogens12060843 (PMC10301873; doi:10.3390/pathogens12060843)
Supplement: Supplementary file 1 [file pathogens-12-00843-s001.zip › pathogens-2291907-supplementary.pdf]

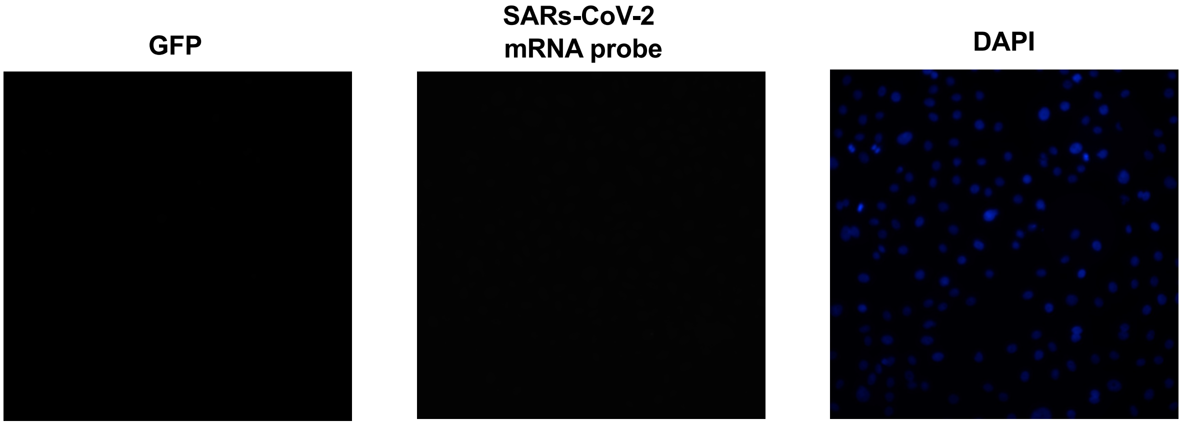

Supplementary Figure 1. smFISH analysis of uninfected oral epithelial cells. Uninfected oral gingival epithelial cells (hTERT TIGKs) were included in the experiment shown in Fig 5. Cells were probed with SARS-CoV-2 mRNA probes by smFISH followed by DAPI stain (blue). The result showed no GFP signal and SARS-CoV-2 mRNA was not detectable in uninfected cells.

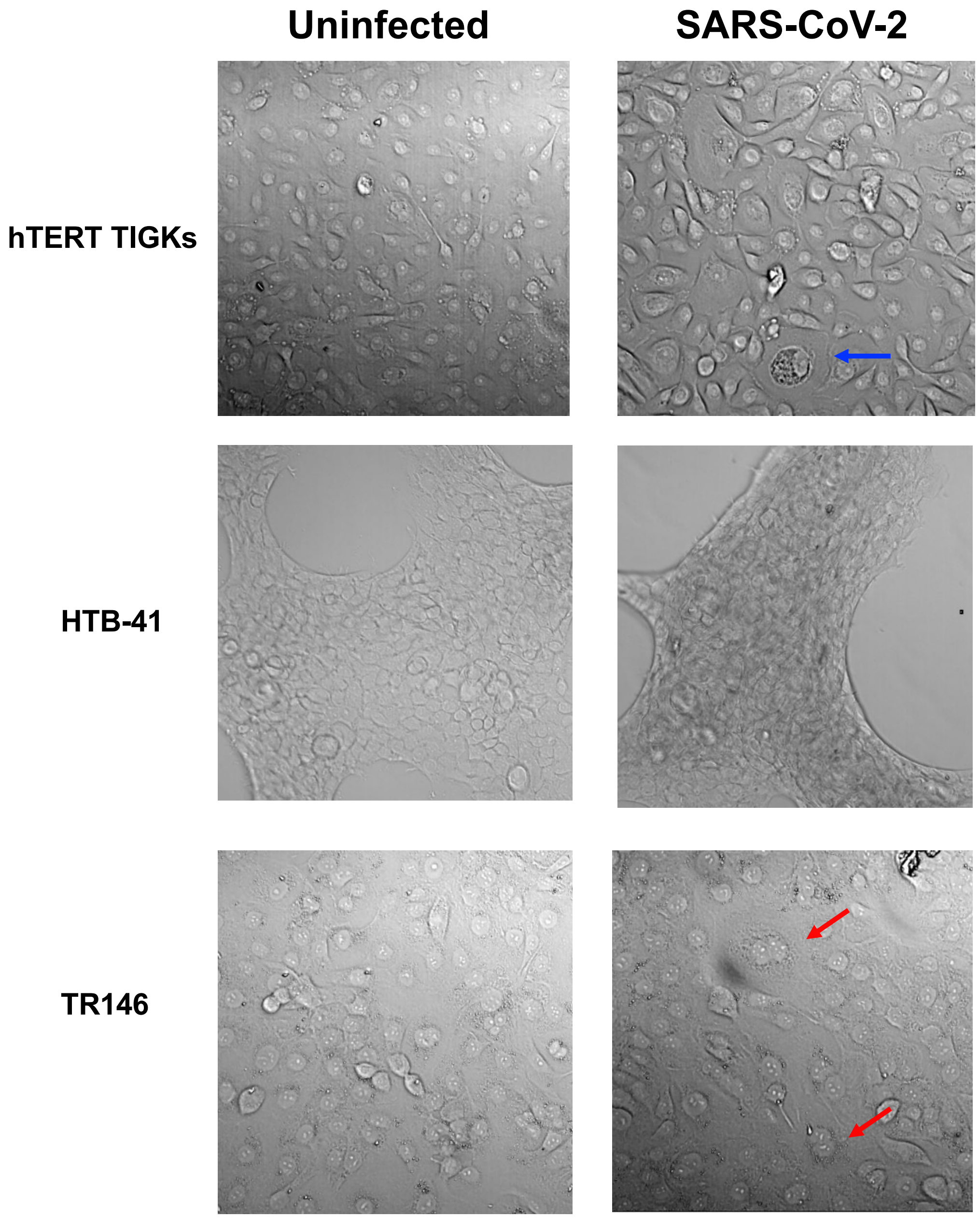

Supplementary Figure 2. Brightfield images of oral epithelial cells with or without SARS-CoV-2 infection. Oral gingival epithelial cells (hTERT TIGKs), salivary gland epithelial cells (A-253), and oral buccal epithelial cells (TR146) were infected by SARS-CoV-2 Wuhan strain expressing GFP as described in Fig 5. Blue and red arrows indicated the altered cell morphology. The images were taken at 48 h p.i. on a Zeiss Axiovert M200 microscope with a 20X objective.
